# Spatial and Temporal Profiles of Growth Factor Expression during CNS Demyelination Reveal the Dynamics of Repair Priming

**DOI:** 10.1371/journal.pone.0022623

**Published:** 2011-07-27

**Authors:** Viktoria Gudi, Jelena Škuljec, Özlem Yildiz, Konstantin Frichert, Thomas Skripuletz, Darius Moharregh-Khiabani, Elke Voß, Kirsten Wissel, Sabine Wolter, Martin Stangel

**Affiliations:** 1 Department of Neurology, Hannover Medical School, Hannover, Germany; 2 Center for Systems Neuroscience, Hannover, Germany; 3 Department of Otolaryngology, Hannover Medical School, Hannover, Germany; 4 Department of Pharmacology, Hannover Medical School, Hannover, Germany; Julius-Maximilians-Universität Würzburg, Germany

## Abstract

Demyelination is the cause of disability in various neurological disorders. It is therefore crucial to understand the molecular regulation of oligodendrocytes, the myelin forming cells in the CNS. Growth factors are known to be essential for the development and maintenance of oligodendrocytes and are involved in the regulation of glial responses in various pathological conditions. We employed the well established murine cuprizone model of toxic demyelination to analyze the expression of 13 growth factors in the CNS during de- and remyelination. The temporal mRNA expression profile during demyelination and the subsequent remyelination were analyzed separately in the corpus callosum and cerebral cortex using laser microdissection and real-time PCR techniques. During demyelination a similar pattern of growth factor mRNA expression was observed in both areas with a strong up-regulation of NRG1 and GDNF and a slight increase of CNTF in the first week of cuprizone treatment. HGF, FGF-2, LIF, IGF-I, and TGF-ß1 were up-regulated mainly during peak demyelination. In contrast, during remyelination there were regional differences in growth factor mRNA expression levels. GDNF, CNTF, HGF, FGF-2, and BDNF were elevated in the corpus callosum but not in the cortex, suggesting tissue differences in the molecular regulation of remyelination in the white and grey matter. To clarify the cellular source we isolated microglia from the cuprizone lesions. GDNF, IGF-1, and FGF mRNA were detected in the microglial fraction with a temporal pattern corresponding to that from whole tissue PCR. In addition, immunohistochemical analysis revealed IGF-1 protein expression also in the reactive astrocytes. CNTF was located in astrocytes. This study identified seven different temporal expression patterns for growth factors in white and grey matter and demonstrated the importance of early tissue priming and exact orchestration of different steps during callosal and cortical de- and remyelination.

## Introduction

In demyelinating diseases of the central nervous system (CNS) like multiple sclerosis (MS) and the leukodystrophies, repair mechanisms and remyelination fail leading to neurological impairment. Regenerative therapies in these diseases are currently not available, thus the understanding of the molecular events during de- and remyelination is necessary to develop new treatment strategies. In recent years a number of growth factors (GFs) has been characterized to be involved in the pathology of MS [1], [2]. Neurotrophins, neuropoietic cytokines, and other growth factors are suggested to support migration, proliferation, and differentiation of glial cells and to regulate myelin synthesis [3], [4], [5]. A disturbed balance of interacting GFs that regulate differentiation of oligodendrocytes and onset of myelination may contribute to the limited remyelination of MS plaques [6], [7]. However, the detailed expression pattern of GF expression during de- and remyelination is not available. Based on *in vitro* and *in vivo* studies basic fibroblast growth factor (FGF-2) and platelet-derived growth factor alpha (PDGF-A) are postulated to promote proliferation and to inhibit differentiation of oligodendrocyte precursor cells (OPCs) [8], [9], [10], [11]. In contrast, insulin-like growth factor I (IGF-I), ciliary neurotrophic factor (CNTF), and transforming growth factor-beta 1 (TGF-ß1) are considered to be key modulators of oligodendrocyte differentiation and myelination [12], [13], [14], [15], [16], [17], [18]. Leukemia inhibitory factor (LIF) and CNTF are known to promote survival of oligodendrocytes [19]. Since cortical MS lesions are associated with an intact blood brain barrier (BBB), alleviated infiltration of lymphocytes, mild astrogliosis [20], [21], and a more extensive remyelination capacity [22] the underlying pathophysiological mechanisms for de- and remyelination seem to differ between white and grey matter. Knowledge of the exact regulation and requirements for GF expression during remyelination may allow the design of specific regenerative treatments for demyelinating diseases.

The murine cuprizone model is well established to study experimental de- and remyelination. Cuprizone feeding leads to oligodendrocyte death and a subsequent reversible demyelination in the corpus callosum and cortex [23], [24]. In particular, the reliable and nearly complete remyelination in this model allows to study the mechanisms that lead to successful regeneration. In addition the temporal course and dynamics of de- and remyelination differ in cerebellar and cerebral white and grey matter thus allowing the investigation of regional differences [25]. The aim of this study was to identify key GFs, which are involved in de- and remyelination in CNS white and grey matter.

## Materials and Methods

### Animals, induction of demyelination, tissue preparation

C57BL/6 male mice were obtained from Charles River (Sulzfeld, Germany). Animals underwent routine cage maintenance once a week and were microbiologically monitored according to Federation of European Laboratory Animal Science Associations recommendations [26]. Food and water were available *ad libitum*. All research and animal care procedures were approved by the Review Board for the Care of Animal Subjects of the district government (Lower Saxony, Germany, approval ID 509.6-42502-03/671 and 33.9-42502-04-07/1309) and performed according to international guidelines on the use of laboratory animals.

Demyelination was induced in 8-week-old male C57BL/6 mice by feeding with 0.2% cuprizone (bis-cyclohexanone oxaldihydrazone, Sigma-Aldrich Inc., USA) mixed into a ground standard rodent chow for 4.5 weeks. For remyelination animals were returned to normal chow for an additional 1.5 week.

### Tissue preparation

At different time points (weeks 1, 2, 3, 3.5, 4, and 4.5 for demyelination; weeks 5, 5.5, and 6 for remyelination) mice were sacrificed and perfused via left cardiac ventricle with RNase free phosphate buffered saline (PBS) for gene expression analysis or with 4% paraformaldehyde (PFA) in phosphate buffer (PBS) for immunohistochemistry. A group size of four or five animals was investigated at each time point. For gene expression analysis the brains were removed and immediately embedded in Tissue Tek® Compound (Sacura, USA), frozen in liquid nitrogen and stored at −80°C until use. Under RNase free conditions, serial coronal sections (bregma +0.98 to −2.46) [27] with a thickness of 30 µm were cut at −20°C. The sections were mounted on polyethylene-naphthalate (PEN) membrane slides (Carl Zeiss MicroImaging GmbH, Germany), fixed for 2 min in 70% ice-cold ethanol, rinsed with DEPC (diethylpyrocarbonat) treated water, and stained for 30 s in 1% cresyl violet acetate solution (Sigma-Aldrich, Germany) in 50% ethanol. Afterwards, sections were dehydrated in graded ethanol series (70% and 100% ethanol) and finally air dried for several minutes. All solutions were prepared with DEPC treated water.

For immunohistochemistry brains were postfixed in 4% PFA in PBS at 4°C overnight and paraffin embedded. For light microscopy, 7 µm serial paraffin sections were cut and dried at 37°C overnight. Sections between bregma −0.70 mm and −1.46 mm according to mouse atlas [27] were analysed.

### Laser microdissection

The Palm® MicroBeam System (Carl Zeiss MicroImaging GmbH, Germany) was used to precisely excise the cerebral cortex and medial part of the corpus callosum from coronal brain sections of treated and age matched control mice (see supporting Fig. S1). Dissected brain regions of the corpus callosum and cortex were collected separately with a sterile 21-gauge needle and stored at −80°C until RNA extraction.

### RNA isolation and quantitative RT-PCR

According to the manufacturer's recommendations total RNA was extracted from the microdissected cortex and corpus callosum tissue using the RNeasy®Mini Kit (Qiagen, Germany) and RNeasy® Micro Kit (Qiagen, Germany), respectively. The RNA concentration was measured with a NanoDrop 1000 spectrophotometer (Thermo Fisher Scientific, USA). cDNA was synthesized using the High Capacity cDNA Reverse Transcription Kit (Applied Biosystems, USA). RNA samples from cuprizone treated and age matched control mice (cortex n = 4, corpus callosum n = 5) were processed in parallel under the same conditions. Real-time PCR analysis was performed using the StepOne™ Real-Time PCR System and appropriate TaqMan assays (Applied Biosystems, USA). All primers were intron-spanning (see supporting Table S1). A negative control containing PCR amplification mix without reverse transcribed cDNA template was included for each PCR plate. Gene expressions of nerve growth factor (NGF), brain-derived neurotrophic factor (BDNF), neurotrophin-3 (NT-3), ciliary neurotrophic factor (CNTF), insulin-like growth factor I (IGF-I), neuregulin 1 (NRG1), epidermal growth factor (EGF), basic fibroblast growth factor (FGF-2), glial cell-derived neurotrophic factor (GDNF), platelet-derived growth factor alpha (PDGF-A), leukemia inhibitory factor (LIF), hepatocyte growth factor (HGF), and transforming growth factor-beta 1 (TGF-ß1) were analysed in the corpus callosum and cortex at 9 time points (demyelination phase: weeks 1, 2, 3, 3.5, 4, and 4.5; remyelination phase: weeks 5, 5.5, and 6). The ΔΔCt method was used to determine differences in expression between cuprizone treated and age-matched control mice. Changes in mRNA expression level were calculated after normalization to hypoxanthin phosphoribosyltransferase *(HPRT)*.

### Isolation of microglia

Corpus callosum and cerebral cortex were dissected from whole brains under a binocular microscope using ultra fine blades. Isolation of microglia was performed based on previously published protocols [28], [29], [30]. For mechanical dissociation, cortex and corpus callosum were grinded separately through a tissue homogenizer (Neolab, Germany) and triturated using pasteur pipettes of decreasing diameter. Afterwards the cell suspension was filtered through a 60 µm cell strainer (Millipore, USA). For separation of microglia, cells were pelleted and resuspended in 3.3 ml ice-cold 70% percoll (GE healthcare, Sweden). The 70% percoll was overlayered with 5 ml 35% ice-cold percoll and 3 ml ice-cold PBS. Density gradient centrifugation was performed at 800 g without brake for 25 minutes at 4°C. After centrifugation the myelin containing 0/35 interface was discarded and the microglia containing 35/70 interface was collected and washed with ice-cold PBS.

### Immunohistochemistry

For immunohistochemistry, paraffin embedded sections were de-waxed, re-hydrated, and microwaved for 5 min in 10 mM citrate buffer (pH 6.0), then blocked for 1 h with PBS containing 3% normal goat serum, 0.1% Triton X-100, and incubated with primary antibody at 4°C overnight. The following primary antibodies were used for myelin proteins: proteolipid protein (PLP) (1∶500, mouse IgG2a, Serotec, Germany) and myelin basic protein (MBP) (1∶500, mouse IgG2b, Covance, USA). Anti Nogo-A (1∶750, rabbit polyclonal, Millipore, USA) was used as a marker for mature oligodendrocytes. Activated microglia were detected using lectin ricinus communis agglutinin 1 (RCA-1) (1∶1000, fluorescein coupled, Vector Laboratories, USA). Glial fibrillary acidic protein (GFAP) (1∶200, mouse IgG1, Millipore, USA or rabbit polyclonal, Dako Cytomation, Germany) was selected as a marker for astrocytes. Nestin (1∶200, mouse IgG1, Millipore, USA) was selected to mark neural precursors. Cellular sources of the following growth factors were analysed: CNTF (1∶200, mouse IgG1; Millipore, USA) and IGF-1 (1∶200, mouse IgG1; Millipore, USA). For immunofluorescent double staining the following pairs of primary antibodies were used: CNTF/GFAP, IGF-1/GFAP, Nestin/GFAP. Sections were then incubated for 1 h with the secondary antibodies: goat anti-mouse IgG H+L Alexa-555/Alexa-488 conjugated (1∶500, Molecular Probes, USA) or/and goat anti-rabbit IgG H+L Alexa-555/Alexa-488 conjugated. Slides were mounted with Mowiol (Calbiochem, USA) containing 4′6-diamidino-2-phenylindole (DAPI; Invitrogen, USA).

### Determination of de- and remyelination in the cortex and corpus callosum

The extent of cortical demyelination was studied as described previously [24]. In particular, myelin protein-stained sections for PLP and MBP were scored using a light microscope (Olympus DP 72, Germany). Scoring of myelin was performed by three blinded observers, using a scale from 0 (complete lack of myelin) to 4 (normal myelin). For determination of myelination in the corpus callosum PLP and MBP stained sections were scored on a scale from 0 (complete demyelination) to 3 (normal myelin) under the same conditions [31].

### Statistical analysis

Statistical analysis was performed using one-way analysis of variance (ANOVA) followed by the Fisher-PLSD-test or Dunnett test for post hoc comparison. LIF mRNA was not detected in the control animals in both the corpus callosum and the cortex. The normalization for LIF was done with the data from week 6. All data are given as arithmetic means ± standard error of the mean (SEM). *P* values of the different ANOVAs are given in the results, while group comparisons derived from post hoc analysis are provided in the figures. In the latter cases, significant effects are indicated by asterisks (compared to the preceding time point) or rhombs (compared to control; *^#^ *p*<0.05; **^##^ *p*<0.01; ***^###^ *p*<0.001).

## Results

### De- and remyelination induced by cuprizone feeding

**Figure 1:**
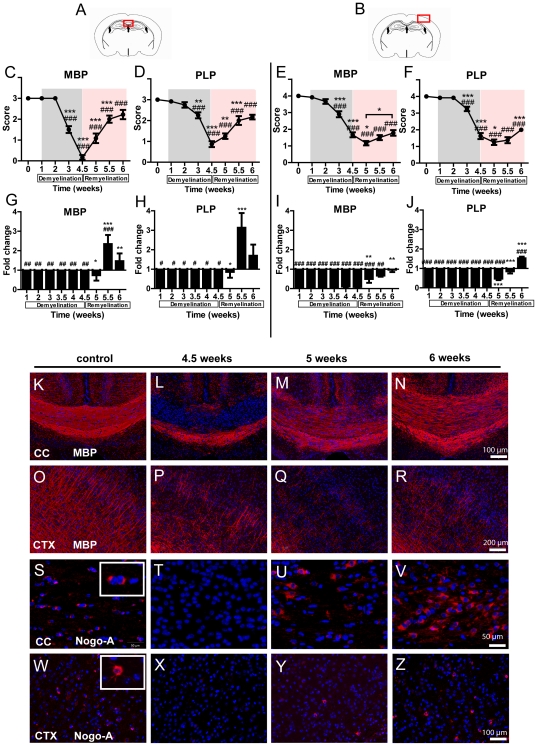
Demyelination and remyelination in the corpus callosum and the cortex. The red marked areas present the investigated middle part of the corpus callosum (**A**) and cortex in (**B**). **C, D**) MBP and PLP myelin protein expression in the corpus callosum. Score 0 represents complete myelin protein loss while score 3 represents normal myelination. **E, F**) MBP and PLP myelin protein expression in the cortex. Score 0 represents complete myelin loss; score of 4 shows normal myelination. **G, H**) mRNA expression of MBP and PLP in the corpus callosum. **I, J**) mRNA expression of MBP and PLP in the cortex. **K–N**) MBP stained sections demonstrate severe demyelination in the corpus callosum (cc) at week 4.5. Remyelination starts already at the week 5 and is almost complete at week 6. **O–R**) MBP stained sections show severe demyelination in the cortex (ctx) at weeks 4.5, and 5. At week 6 the MBP amount increases (MBP is shown in red, nuclear staining with DAPI in blue). Representative sections show Nogo-A positive oligodendrocytes in the corpus callosum (cc) (**S–V**) and cortex (ctx) (**W–Z**). No oligodendrocytes are seen in the corpus callosum and cortex at week 4.5 (**T, X**). Oligodendrocytes reappear at week 5 in both, the corpus callosum and cortex (**U, Y**) (Nogo-A is shown in red, nuclear staining with DAPI in blue).

**Figure 2:**
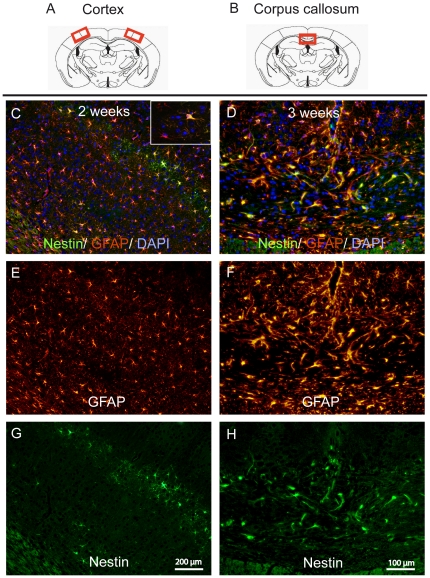
Astrocytic expression of nestin during early demyelination in the corpus callosum and the cortex. The red marked areas represent the investigated medial corpus callosum (**A**) and parts of lateral cortex (**B**). Nestin positive cells (in green) are present at week 2 in the cortex (**G**) and at week 3 in the corpus callosum (**H**). GFAP stained activated astrocytes (in red) are numerously present at week 2 of the cuprizone diet in the cortex (**E**) and at week 3 in the corpus callosum (**F**). Several nestin positive cells are double positive for GFAP in both the cortex (**C**) and the corpus callosum (**D**) (Nestin is shown in green, GFAP in red, nuclear staining with DAPI in blue).

To determine de- and remyelination pattern in response to cuprizone treatment, immunohistochemical stainings and real-time PCR analyses for the myelin proteins PLP and MBP were performed. As demonstrated in figure 1, maximal loss of MBP (Fig. 1C, L) and PLP (Fig. 2D) is present in the corpus callosum after 4.5 weeks of cuprizone feeding (for both myelin proteins p<0.0001). At week 6 (after 1.5 week of normal diet), the immunoreactivity for both MBP (Fig. 1C, N) and PLP (Fig. 1D) was nearly completely recovered in the corpus callosum. The mRNA expression for MBP and PLP was strongly down-regulated already after 1 week and remained at low levels during the following weeks of cuprizone treatment (for both p<0.0001) (Fig. 1G, H). After cuprizone withdrawal from the diet, MBP and PLP mRNA expression returned to normal levels (Fig. 1G, H).

In the cortex, the most severe loss of MBP and PLP was detected at week 5 (p<0.0001) (Fig. 1E, F, Q). The mRNA expression of MBP and PLP was strongly decreased after one week and remained down-regulated during the whole cuprizone feeding (for both p<0.0001) (Fig. 1I, J). The normalisation of mRNA expression for MBP and PLP was achieved at weeks 5.5 and 6, 0.5–1 week later than in the corpus callosum.

### Glial reactions during cuprizone treatment

To follow the oligodendroglial cellular response to cuprizone feeding we used the marker Nogo-A. After 4.5 weeks of cuprizone treatment Nogo-A positive cells were detected neither in the medial corpus callosum nor in the cortex (Fig. 1T, X). At week 5 mature oligodendrocytes reappeared in the corpus callosum (Fig. 1U). In the cortex, Nogo-A positive cells were seen only sporadically at week 5 (Fig. 1Y). At weeks 5.5 and 6 the amount of Nogo-A positive cells further increased in both areas (Fig. 1V, Z).

Accumulation of activated microglia was studied by RCA-1 staining (see supporting Fig. S2). In the corpus callosum RCA-1 positive cells were detected already after 2 weeks of cuprizone feeding. During the following 2.5 weeks the amount of activated microglia increased steadily and reached a peak at week 4.5 concomitant with the demyelination peak. Thereafter, a continuous decrease of activated microglia was observed. In the cortex, microgliosis occurred in an obviously reduced density as compared to the corpus callosum. Within the cortex an infiltration of activated microglia was found at weeks 3–5. At week 6 microglia were only sporadically found in the cortex in the 6^th^ cellular layer.

Astrogliosis was studied by GFAP immunostaining (see supporting Fig. S2). Consistent to our previous results [25], few GFAP positive astrocytes were seen in the corpus callosum in untreated controls, while in the cortex GFAP positive astrocytes were found sporadically. After 2 weeks of cuprizone treatment a strong astrogliosis occurred in the cortex. In the corpus callosum strong astrogliosis was observed with a delay of one week as compared to cortex. In both regions astrogliosis was detectable in the following weeks until week 6.

Nestin positive cells were detected in the cortex at week 2 (Fig. 2G). These cells showed astrocytic shape and were double positive for the astrocytic marker GFAP (Fig. 2C). Interestingly, nestin positive cells were situated mostly in the fourth cellular layer of the cortex. At the following weeks nestin positive cells were sporadically seen in the cortex, mainly in the fifth and sixth cellular level. In the corpus callosum nestin positive cells occurred only sporadically at week 2. The main amount of these cells was observed at weeks 3 (Fig. 2H), 4, and 4.5 followed by a gradual decrease. Again, these cells showed astrocytic shape and were double positive for the astrocytic marker, GFAP (Fig. 2D).

### Analysis of growth factor mRNA expression profiles during de- and remyelination in the corpus callosum and cortex

**Figure 3:**
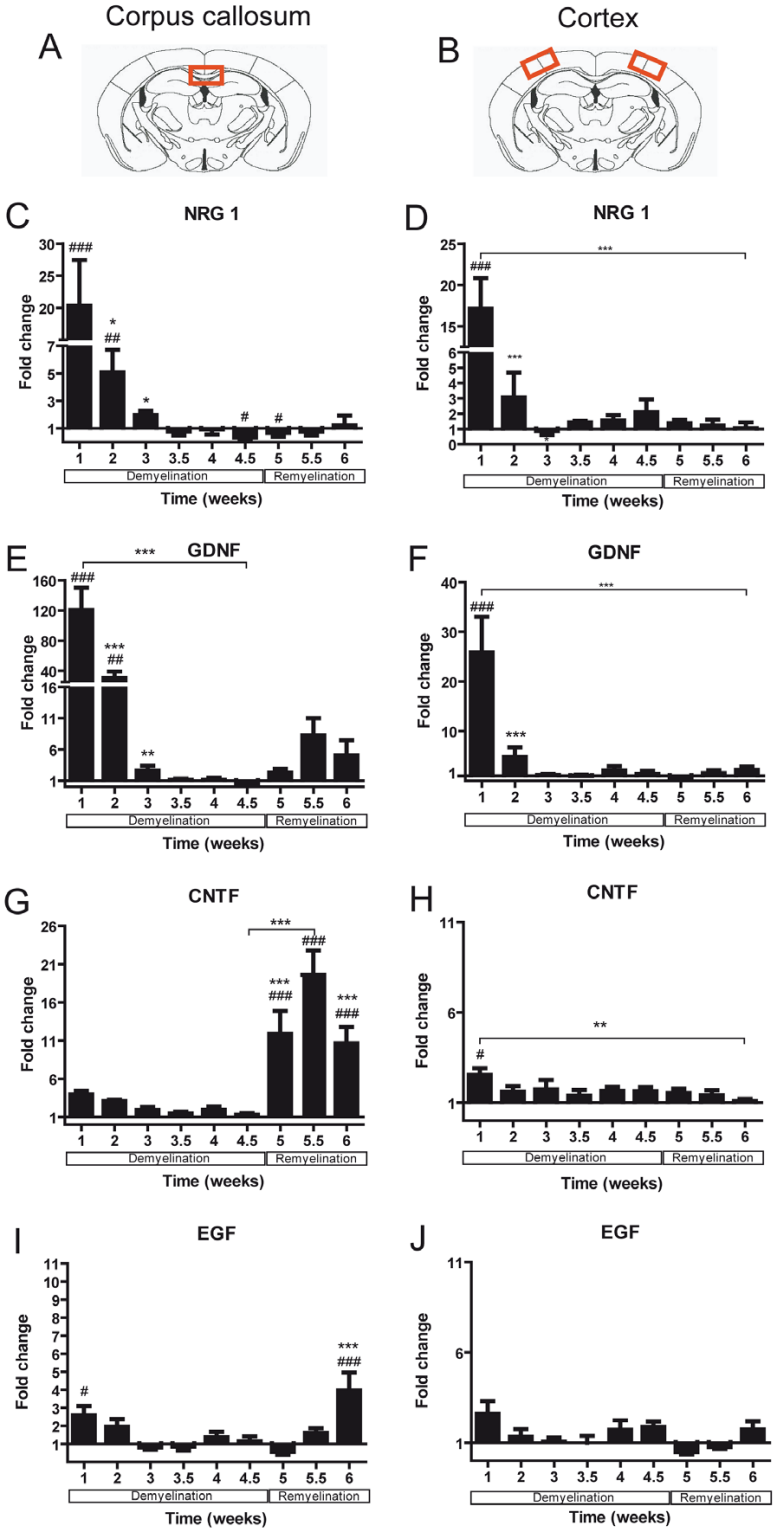
Expression of growth factor mRNA in the corpus callosum and cortex of mice undergoing demyelination / remyelination. The red marked areas present the investigated middle part of the corpus callosum in **A** and cortex in **B**. **C–J**) Graphs show mRNA expression fold changes of NRG1, GDNF, CNTF, and EGF mRNA expression in the corpus callosum (**C–I**) and in the cortex (**D–J**) compared to age-matched controls and normalized with *HPTR* using the ΔΔCt method. Significant changes are indicated by rhombs (compared to control) or asterisks (compared to the preceding time point) (*^#^ *p*<0.05; **^##^ *p*<0.01; ***^###^ *p*<0.001).

**Figure 4:**
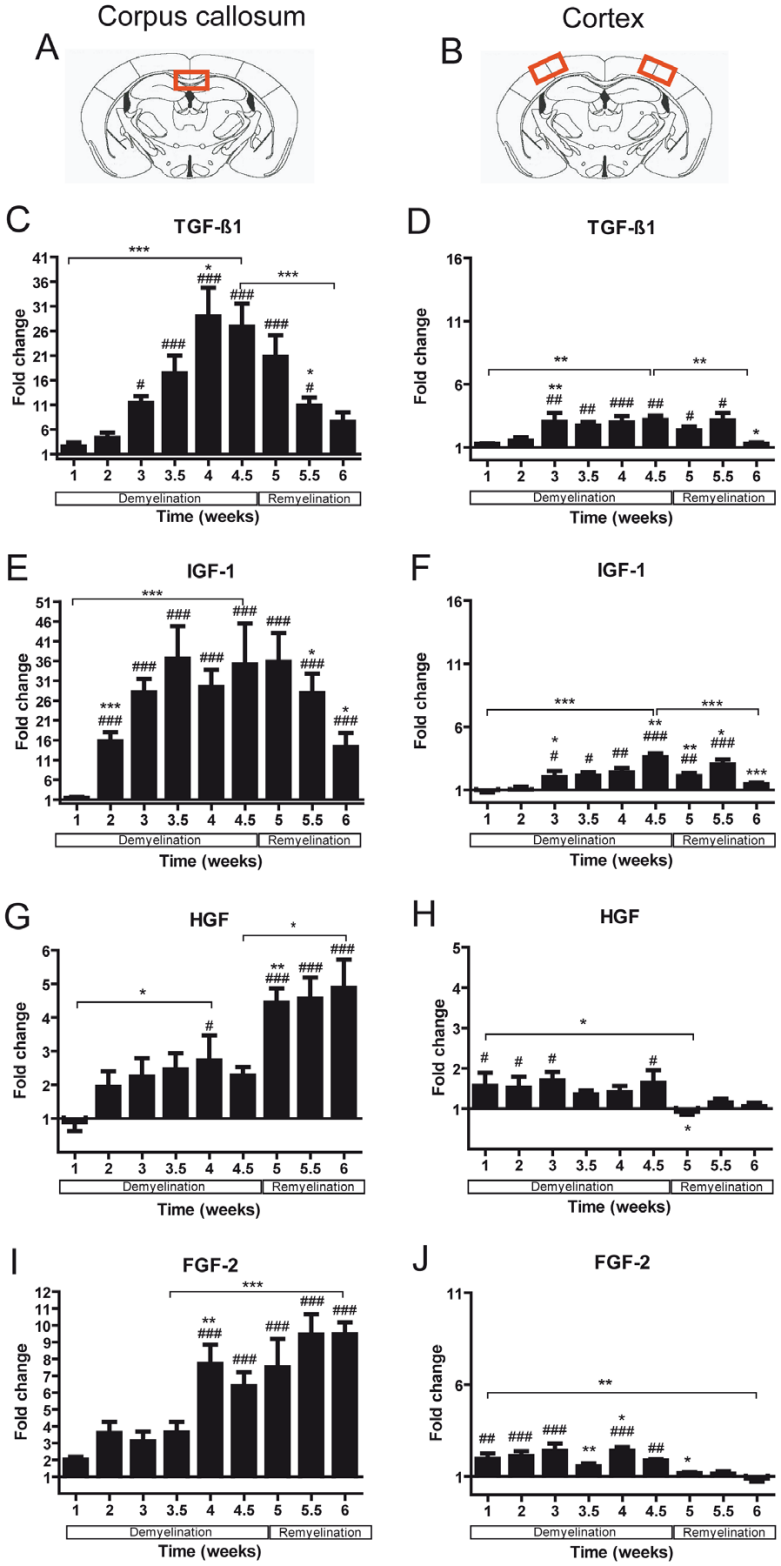
Expression of growth factor mRNA in the corpus callosum and the cortex of mice undergoing demyelination / remyelination. The red marked areas present the investigated middle part of the corpus callosum (**A**) and the cortex (**B**). **C–J**) Graphs show mRNA expression fold changes of TGF-ß1, IGF-1, HGF, and FGF-2 mRNA expression in the corpus callosum (**C–I**) and in the cortex (**D–J**) compared to the age-matched controls and normalized with *HPRT* using the ΔΔCt method. Significant effects are indicated by rhombs (compared to control) or asterisks (compared to the preceding time point) (*^#^ *p*<0.05; **^##^ *p*<0.01; ***^###^ *p*<0.001).

**Figure 5:**
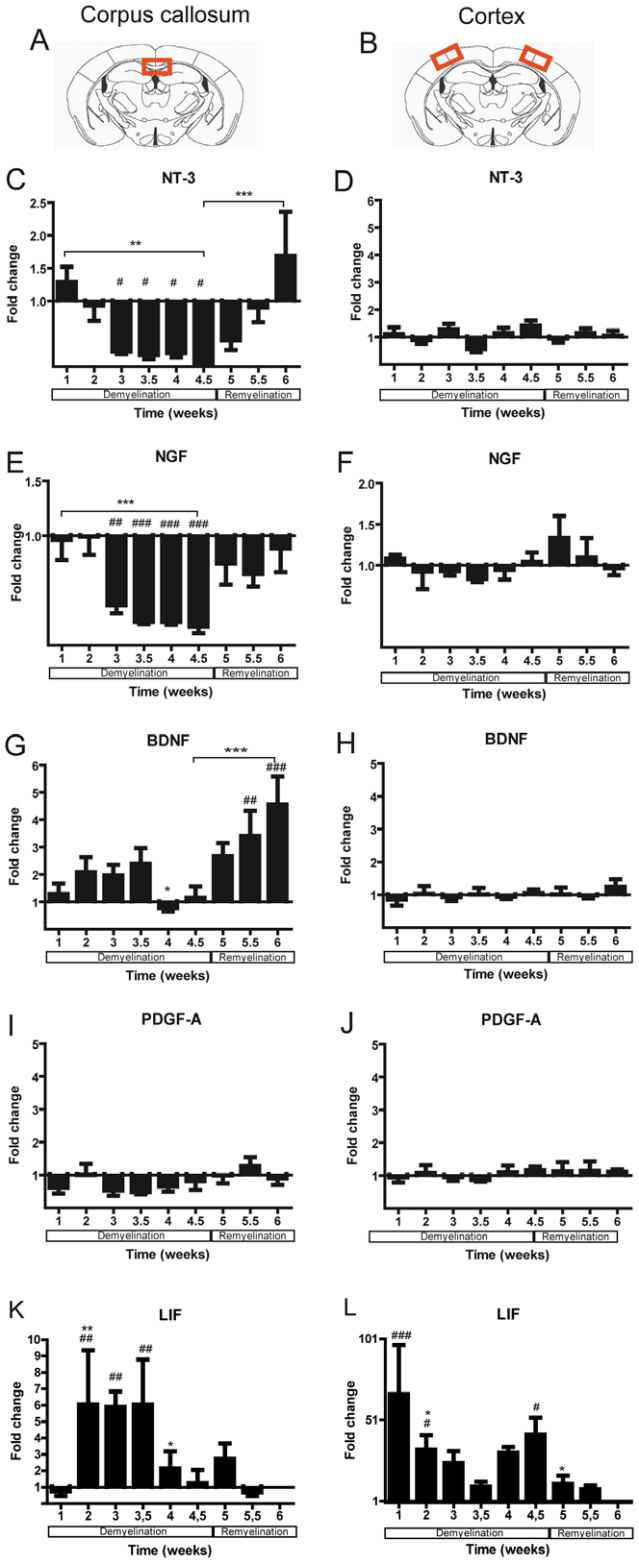
Expression of growth factor mRNA in the corpus callosum and the cortex of mice undergoing demyelination / remyelination. The red marked areas present the investigated middle part of the corpus callosum (**A**) and the cortex (**B**). **C–L**) Graphs show mRNA expression fold changes of NT-3, NGF, BDNF, PDGF-A, and LIF mRNA expression in the corpus callosum (**C–K**) and in the cortex (**D–L**) compared to the age-matched controls and normalized with *HPRT* using the ΔΔCt method. Significant effects versus controls are indicated by rhombs or asterisks if compared to the preceding time point (*^#^ *p*<0.05; **^##^ *p*<0.01; ***^###^ *p*<0.001).

To identify growth factors produced in the white and grey matter during de- and remyelination, mRNA expression of thirteen growth factors was studied in the medial corpus callosum and the lateral cerebral cortex. The individual profiles of each growth factor mRNA expression in the white and grey matter are shown in figures 3, 4, and 5.

NRG1 and GDNF mRNA expression was massively up-regulated after the first week of cuprizone feeding in both the corpus callosum and cortex (for both p<0.0001). During the following weeks NRG1 and GDNF mRNA expression normalized (Fig. 3C, D, E, F). At the onset of remyelination, there was a trend for an increased amount of GDNF mRNA expression in the corpus callosum that failed to be significant.

CNTF and EGF mRNA production showed significant changes mostly in the white matter (corpus callosum: for both p<0.0001; cortex: for CNTF p = 0.01; EGF p = 0.05). Both factors were slightly up-regulated after the first week of cuprizone feeding (CNTF - 4 times in the corpus callosum and 2.5 times in the cortex; EGF - 2.5 times in the corpus callosum and 3.6 times in the cortex). During the remyelination phase CNTF and EGF mRNA were strongly elevated only in the white matter (Fig. 3G, H, I, J).

TGF-ß1 and IGF-1 mRNA expression increased significantly after cuprizone treatment and reached their peak after 4.5 weeks at peak demyelination in both investigated areas (for both p<0.0001). With progressing remyelination, mRNA expression of these two factors decreased gradually (Fig. 4C, D, E, F).

The mRNA synthesis of HGF and FGF-2 was strongly regulated in both white and grey matter during the whole experimental time window (corpus callosum: for both factors p<0.0001; cortex: FGF-2 p<0.0001; HGF p = 0.05) (Fig. 4G, H, I, J). Whereas in the cortex FGF-2 mRNA was increased at a constant level during the whole demyelination, in the corpus callosum there was a peak of mRNA elevation at weeks 4 and 4.5. During remyelination the mRNA of HGF and FGF-2 continued to be elevated in the corpus callosum. In contrast, in the cortex the mRNA expression of these two factors was comparable to control levels.

The mRNA expression of NT-3 and NGF decreased significantly upon cuprizone treatment (for both p<0.0001) in the corpus callosum (Fig. 5C, E). With the cessation of the cuprizone diet NT-3 and NGF mRNA expression returned to normal levels.

BDNF mRNA synthesis was only slightly elevated in the corpus callosum during the first 3.5 weeks of cuprizone feeding, and normalized within the following weeks. During remyelination the mRNA expression of BDNF increased significantly (p = 0.001) in the corpus callosum (Fig. 5G).

In the cortex, the mRNA expression of NT-3, NGF, and BDNF did not change significantly and persisted on levels comparable to controls during both de- and remyelination (Fig. 5D, F, H).

The PDGF-A mRNA synthesis was not changed during de- and remyelination in both regions (Fig. 5I, J).

The expression of LIF mRNA was hardly detectable in both the corpus callosum and the cortex of control mice. Thus, these mRNA expressions were calculated in comparison to the mRNA expression at week 6. In the white matter, LIF mRNA was particularly increased at the weeks 2 and 3 of cuprizone feeding (p = 0.005) (Fig. 5K). In the grey matter, LIF mRNA synthesis was strongly increased during the first weeks (p = 0.002) and at the peak of demyelination (Fig. 5L).

### Seven different temporal patterns of growth factor mRNA expression are identified in the white and grey matter

**Figure 6:**
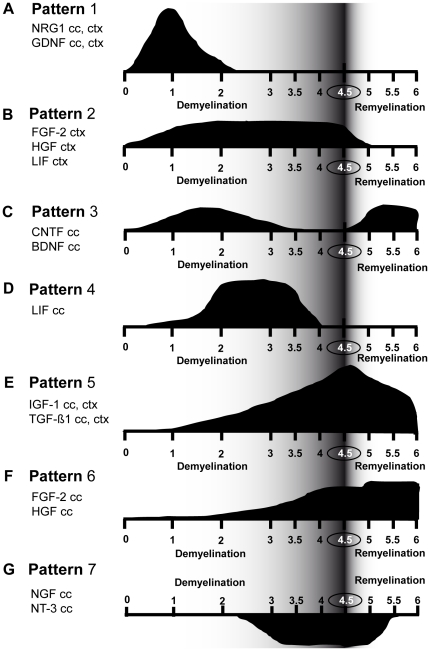
Different temporal pattern of growth factors mRNA expression. **A**) Pattern 1: characteristic for NRG1 and GDNF mRNA expression in the corpus callosum and the cortex. **B**) Pattern 2: characteristic for FGF-2, HGF, and LIF mRNA expression in the cortex. **C**) Pattern 3: characteristic for CNTF, BDNF mRNA expression in the corpus callosum. **D**) Pattern 4: characteristic for LIF mRNA expression in the corpus callosum. **E**) Pattern 5: characteristic for IGF-1 and TGF-ß1 mRNA expression in both the corpus callosum and the cortex. **F**) Pattern 6: characteristic for FGF-2 and HGF mRNA expression in white matter. **G**) Pattern 7: characteristic for NGF and NT-3 mRNA expression in the corpus callosum.

In order to summarise the data, we identified 7 different temporal pattern of mRNA expression for all studied growth factors in the white and grey matter (Fig. 6).

Pattern 1: The mRNA expression was strongly up-regulated during the first two weeks and is nearly at control level during the following weeks of de-and remyelination. This pattern was typical for GDNF and NRG1 mRNA expression in both white and grey matter (Fig. 6A).

Pattern 2: The mRNA expression was increased during the whole demyelination phase and normalises with the onset of remyelination. This pattern was identified only in the cortex for FGF-2, HGF, and LIF (Fig. 6B).

Pattern 3: The mRNA expression was elevated during early demyelination (1–3 weeks of cuprizone feeding), then returns to control levels and has a second peak up-regulation during remyelination. This pattern was characteristic for CNTF and BDNF in the corpus callosum (Fig. 6C).

Pattern 4: The mRNA expression was up-regulated only between weeks 2 and 3.5. This pattern was seen only for LIF in the corpus callosum (Fig. 6D).

Pattern 5: mRNA expression increased gradually during the whole demyelination period reaching its maximum at the peak of demyelination followed by a decline during remyelination. This pattern was characteristic for IGF-1 and TGF-ß1 in the white and grey matter (Fig. 6E).

Pattern 6: mRNA expression increased during late demyelination and stays elevated during remyelination. This pattern was determined for FGF-2 and HGF in the corpus callosum (Fig. 6F).

Pattern 7: mRNA expression was down-regulated during peak demyelination and normalised after the onset of remyelination. This pattern was identified for NT-3 and NGF in the corpus callosum (Fig. 6G).

### mRNA expression of GDNF, IGF-1 and FGF-2 was elevated in the microglia fraction

**Figure 7:**
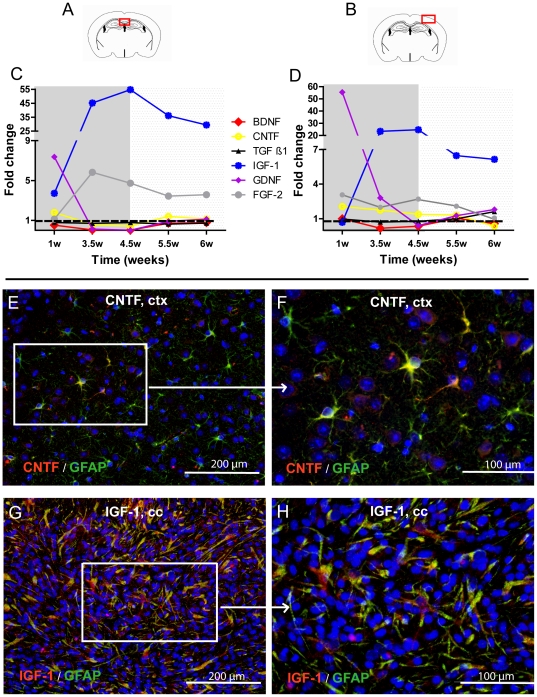
Glial localization of growth factor production. The expression of the growth factors IGF-1, FGF-2, CNTF, GDNF, BDNF, and TGF-ß1 was analysed in microglia by real-time PCR. Microglia were isolated from the cortex (ctx) and corpus callosum (cc) and RNA was extracted from the controls and cuprizone treated mice after 1, 3, 4.5, 5.5, and 6 weeks. Fold changes of mRNA levels of the different growth factors are presented after normalization to *HPRT* and compared to the expression of untreated mice ( = 1, showed by the dotted line). Representative pictures show the CNTF (red) expression by GFAP (green) expressing astrocytes in the cortex at the week 2. Double positive cells are yellow (**E, F**). Representative pictures show the IGF-1 (red) expression by GFAP (green) expressing astrocytes in the corpus callosum at week 4.5 (**G, H**).

To clarify whether microglia could provide growth factors relevant for the regulation of cuprizone induced de- and remyelination, we analysed the expression of IGF-1, TGF-ß1, FGF-2, BDNF, GDNF, and CNTF by quantitative PCR after isolation of microglia from cortex and corpus callosum. The expression of growth factors was measured in microglia isolated from untreated mice and cuprizone treated mice after 1, 3.5, 4.5, 5.5, and 6 weeks. The GDNF mRNA expression was strongly elevated only after the first week of cuprizone feeding in both the corpus callosum and the cortex (Fig. 7C, D). A strong up-regulation of IGF-1 was found in cortical and callosal microglia reaching a maximum after 3.5–4.5 weeks. During remyelination, the expression of IGF-1 was further up-regulated in both the white and grey matter showing however a tendency to decline. The FGF-2 mRNA expression was slightly elevated during demyelination in the cortex (Fig. 7D). In the corpus callosum, FGF-2 mRNA expression reached its maximum during severe demyelination after 3.5 weeks of cuprizone feeding (Fig. 7C) and decreased after the onset of remyelination. The expression of BDNF and CNTF was neither changed in the cortex nor in the corpus callosum (Fig. 7C, D).

### IGF-1 and CNTF are produced in astrocytes

Immunochemical stainings for IGF-1 and CNTF were performed in order to identify the cellular source of these growth factors. The cells producing IGF-1 as well CNTF showed astrocytic shape. The double stainings with GFAP confirmed the astrocytic localisation of these growth factors (Fig. 7E–H).

## Discussion

Growth factors are involved in the orchestration of proliferation, migration, and differentiation of glial cells in demyelinating diseases and are suggested to support remyelination in the CNS. Using the murine cuprizone model of toxic demyelination we analysed the expression of various neurotrophic factors during demyelination and the subsequent remyelination in the white and the grey matter and identified seven different temporal expressions pattern.

After one week of cuprizone feeding mature oligodendrocytes become apoptotic [32], while after four weeks they are nearly completely depleted. As a consequence of this we observed a severe loss of myelin proteins in both the corpus callosum and cortex. Thereafter, oligodendrocytes began to reappear. The temporal myelination pattern was different between corpus callosum and cortex and the maximum of demyelination occurred delayed in the cortex, as previously described [25]. Subsequently, cortical reexpression of myelin proteins was also delayed. Along with demyelination, microglial infiltration and astrogliosis were observed in both regions.

Since exogenous CNTF induces reactive astrogliosis and the up-regulation of GFAP mRNA [33], CNTF has been suggested to be a key player in astrogliosis. In our work CNTF mRNA expression was up-regulated in the first two weeks of cuprizone feeding in both the corpus callosum and cortex and may account for the astrogliosis. Interestingly, GDNF and NRG1 were strongly up-regulated in the white and grey matter at the first two weeks of cuprizone feeding, when demyelination was not yet detectable. GDNF was shown to be up-regulated in astrocytes during various pathophysiological conditions [34], [35], [36] and proliferative effects on C6 glioma cells [37] have been demonstrated. Thus, the up-regulation of GDNF may provide an activating signal for astrocytes. Furthermore, GDNF was also shown to stimulate microglial activation and to be produced by microglia [38], [39], [40]. In our study microglia were identified as the source of GDNF but not NRG1. Activated microglia were first seen after 2 weeks of cuprizone feeding in both corpus callosum and cortex. There was an up-regulation of LIF mRNA at weeks 2–3 in the corpus callosum, while in the cortex LIF was continuously up-regulated during the whole demyelination phase. Since LIF is known to modulate ongoing inflammatory responses by enhancing the proliferation, activation, and migration of microglia/macrophages and stimulates the phagocytosis of myelin debris [41], [42], [43] we hypothesise that LIF may also modulates microglial activity in this model.

IGF-1 and TGF-ß1 mRNA expression were similar in both studied regions and showed a gradual elevation during demyelination with a peak at week 4.5 followed by a gradual decrease during remyelination. These results are in line with data generated in other models of experimental demyelination [44], [45] and in cuprizone induced demyelination [46]. However, in our study the expression of IGF-1 was examined separately in the corpus callosum and the cortex, whereas Mason et al. studied tissue from whole brains. In addition, we investigated the source of IGF-1 mRNA expression and found an elevation of IGF-1 expression in both microglia and in reactive astrocytes.

In the cortex an increased expression of FGF-2 and HGF mRNA was observed during demyelination, which returned to normal levels during remyelination. In contrast, in the corpus callosum an elevated expression of FGF-2 and HGF mRNA persisted during remyelination. Microglia were identified as a source of FGF-2. It is widely accepted that the main impact of FGF-2 is the support of OPC proliferation [8] and inhibition of progenitor differentiation/myelination [47], [48]. Using *in situ*-hybridization methods, FGF-2 has been shown to be up-regulated after 6 weeks of 0.3% cuprizone feeding in the corpus callosum [10]. It should be noted that the treatment with 0.3% cuprizone induces a different demyelination pattern as compared to 0.2% cuprizone (31). In our study mice were fed with 0.2% cuprizone and we found a strong up-regulation of FGF-2 mRNA after 4.5 weeks (timepoint of maximal myelin loss) corresponding to the intensive proliferation of OPCs. It has been shown previously that the absence of FGF-2 promotes regeneration of oligodendrocytes after cuprizone induced demyelination [10]. We therefore assume that the down-regulation of FGF-2 in the cortex allows OPC differentiation and subsequent myelin formation. However, in the corpus callosum a different expression of FGF-2 was observed. An up-regulation of FGF-2 during remyelination has also been found in lysolecithin induced demyelination [44] as well as *in vitro* in myelinating aggregate cultures [49]. It can only be speculated that FGF-2 possesses multiple functions and acts not only directly on oligodendrocytes but also influences other cell types promoting myelination indirectly. Moreover, different splice variants of FGF-2 could have different modes of actions or FGF-2 could act through different receptors.

HGF was shown to be chemotactic for OPCs *in vitro* and a functional HGF/c-Met system, which can influence the proliferation, development, and cytoskeletal organization, is present in oligodendrocytes [50], [51]. HGF producing microglia/macrophages were present in EAE lesions during the recovery phase, but not in the acute stage of disease [51], suggesting to play a role in remyelination.

Interestingly, CNTF and BDNF showed a second peak in mRNA expression at the onset of remyelination in the corpus callosum, but not in the cortex. CNTF has been detected in astrocytes in the remyelinating phase after viral-induced spinal cord demyelination [52]. In contrast, in the lysolecithin model CNTF mRNA expression was not up-regulated during remyelination [44]. The conflicting results of CNTF expression in different demyelination models and CNS regions may be due to the heterogeneity of OPC populations and involvement of peripheral inflammatory cells. In our study CNTF mRNA up-regulation corresponded with the elevation of MBP and PLP mRNA expression in the corpus callosum. We hypothesize that CNTF may promote remyelination of the corpus callosum but not of the cortex. Similarly, CNTF was shown to promote OPCs differentiation in optic nerve, but it is not supportive for differentiation of cortical OPCs [53]. In addition, CNTF and CNTF receptor alpha expression patterns differ between white and grey matter astrocytes [54]. This may be again due to the heterogeneity of astrocytes and OPCs in different CNS areas. Since cortical OPCs do not express the full-length BDNF receptor, trkB, BDNF can not promote differentiation of cortical oligodendrocytes [55]. Probably, cortical OPCs do not require BDNF support for differentiation, which could explain the observed differences in BDNF mRNA expression between the white and grey matter.

The expression of NT-3 and NGF mRNA was significantly down-regulated during demyelination and restored at the onset of remyelination in the corpus callosum. The mRNA expression of all analysed neurotrophins did not change during the whole experiment, indicating that these neurotrophins do not to play a crucial role during de- or remyelination in the cortex. NT-4 was not detected in both areas, neither during de- nor during remyelination.

Taking our results and the published data in consideration, the following scenario of de- and remyelination orchestration in the corpus callosum and cortex could be suggested: Demyelination is initiated already after 1 week of cuprizone feeding. NRG1, GDNF, and CNTF are suggested to induce astrogliosis and microgliosis. In turn, reactive astrocytes produce LIF that further stimulates migration, activation, and proliferation of microglia. Activated microglia and reactive astroglia prepare remyelination by the release of IGF-1, FGF-2, and HGF, which support migration, proliferation, and differentiation of OPCs. CNTF, FGF-2, BDNF, and HGF are suggested to be the key player in promoting remyelination in the corpus callosum. In the cortex, these latter factors may not be required or act in different time windows.

Interestingly, some growth factors showed similar temporal expression pattern arguing for either a synergetic mode of action or for a certain degree of redundancy. According to the temporal expression profile we could identify seven different pattern of mRNA expression in the white and grey matter. These patterns demonstrate the requirement of different factors during the various stages of de- and remyelination to achieve successful repair. It is likely that this orchestration of repair promoting factors follows a strict pattern since this can also be observed in the lysolecithin model of demyelination, in particular for IGF-1, FGF-2, TGF-ß1 [44]. Hence, it seems unlikely that our results represent a cuprizone-specific epiphenomenon. However, since demyelination occurs rapidly in the lysolecithin model there is no early priming of the lesions as in the cuprizone model demonstrated here.

In conclusion, this detailed longitudinal analysis of GF expression during de- and remyelination reveals seven different pattern suggesting that there is an early priming of tissue reactivity even before demyelination is visible that probably already initiates repair processes. The results also demonstrate that during each phase of tissue destruction and repair there is a requirement for a complex and exact orchestration of different signals which are also region-specific in the CNS. Besides astrocytes, intrinsic microglia also provide some of these signals.

## Supporting Information

Figure S1 **Laser microdissection.** **A**) microdissected area of the corpus callosum; **B**) microdissected area of the cortex; **C**) Overview of coronal section with the dissected corpus callosum and cortex. Brain sections were stained with cresyl violet.
(TIF)

Figure S2 **Microglia and astrocytes during de- and remyelination in the corpus callosum and the cortex.** Representative sections show microglia in the corpus callosum (cc) (**A–D**) and the cortex (ctx) (**E–H**), stained with fluorescein coupled anti-RCA-1 (in green). The peak of microgliosis was observed at week 4.5 in both the corpus callosum and the cortex (**B, F**). Astrogliosis is shown in the corpus callosum (**I–L**) and the cortex (**M–P**), stained with anti-GFAP and Alexa 555 secondary antibodies (in red). In untreated animals numerous GFAP positive cells are found in the corpus callosum (**I**) in contrast to only few GFAP positive cells in the cortex (**M**). Upon cuprizone treatment reactive astroglia appear in the cortex and in the corpus callosum. At week 4.5 hypertrophic astrocytes are abundantly detected in both areas (**J, N**). At weeks 5 and 6 astroglia are still presented in large numbers in the corpus callosum and the cortex, however, the shape of astrocytes alters and their processes become thinner (**K, L, O, P**). For nucleus staining, slides were counterstained by DAPI.
(TIF)

Table S1 **TaqMan® Gene Expression Assays (Applied Biosystems, USA) were used to investigate mRNA expression of different growth factors.**
(DOC)
