# Supplementary figures and images for: Spatial and Temporal Profiles of Growth Factor Expression during CNS Demyelination Reveal the Dynamics of Repair Priming
Source: PLoS One. 2011 Jul 27;6(7):e22623. doi: 10.1371/journal.pone.0022623 (PMC3144923; doi:10.1371/journal.pone.0022623)

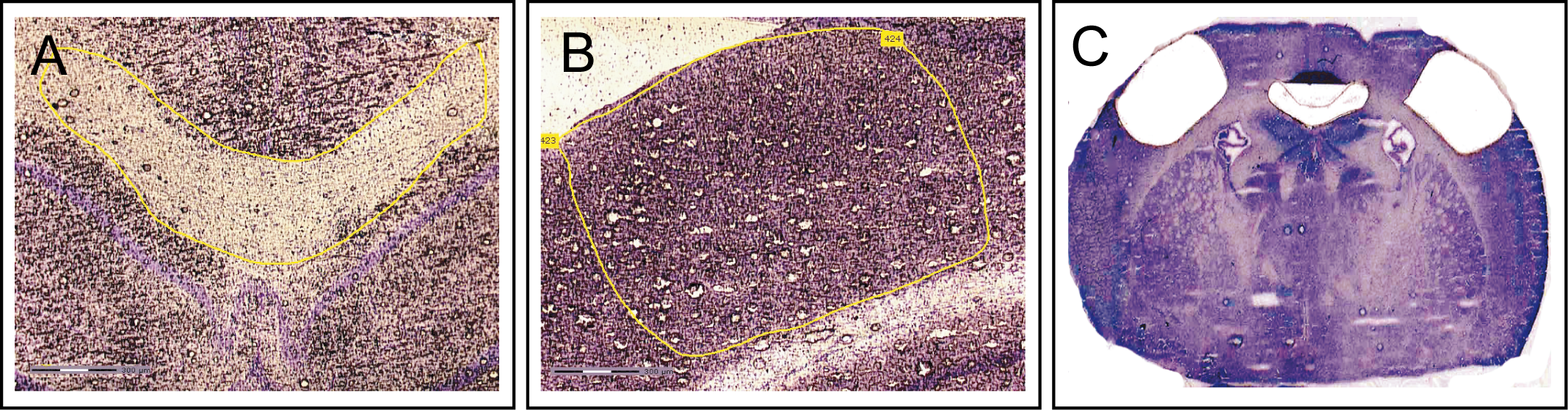

Supplement: Figure S1 — Laser microdissection. A) microdissected area of the corpus callosum; B) microdissected area of the cortex; C) Overview of coronal section with the dissected corpus callosum and cortex. Brain sections were stained with cresyl violet. (TIF) [file pone.0022623.s001.tif]

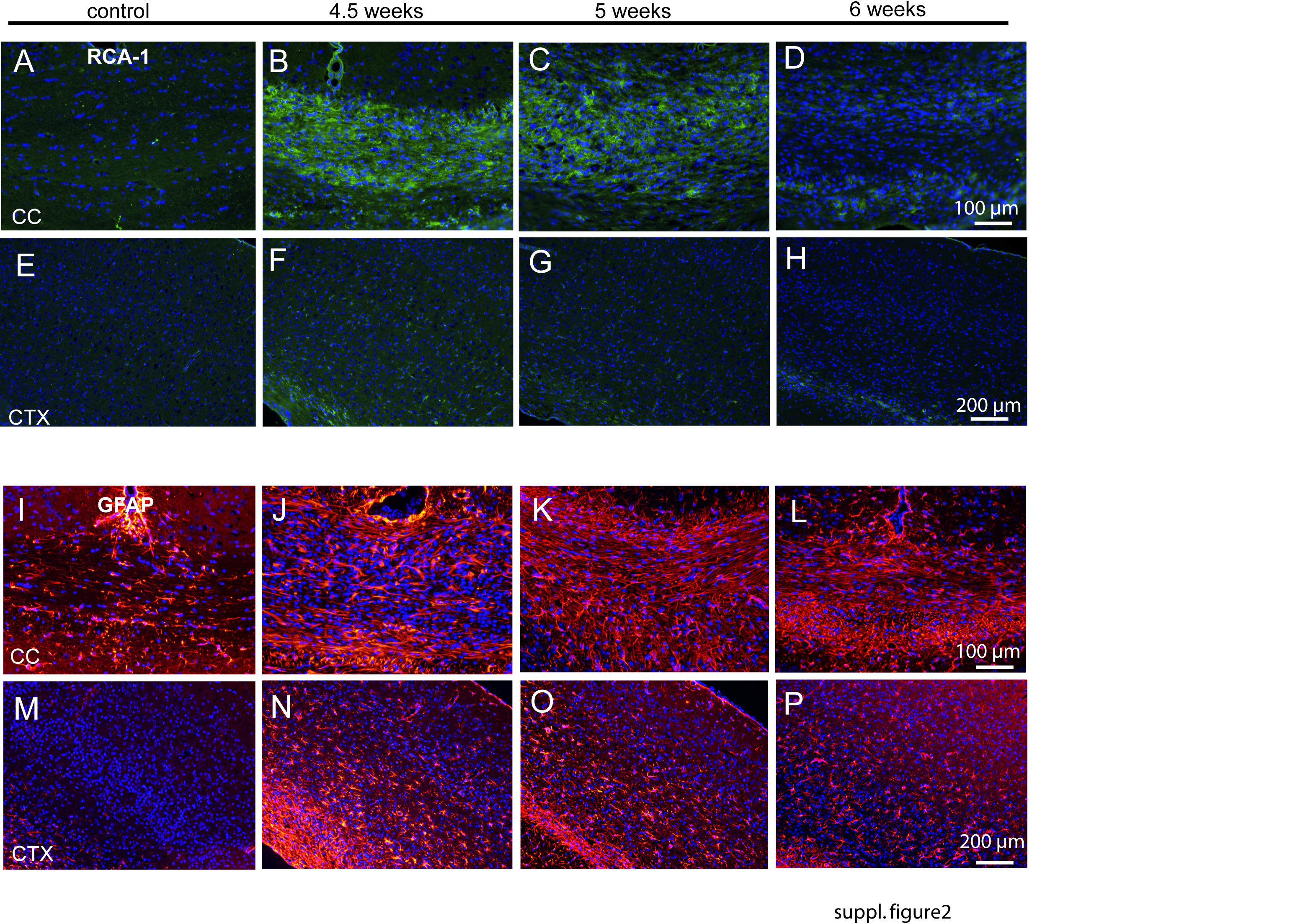

Supplement: Figure S2 — Microglia and astrocytes during de- and remyelination in the corpus callosum and the cortex. Representative sections show microglia in the corpus callosum (cc) (A–D) and the cortex (ctx) (E–H), stained with fluorescein coupled anti-RCA-1 (in green). The peak of microgliosis was observed at week 4.5 in both the corpus callosum and the cortex (B, F). Astrogliosis is shown in the corpus callosum (I–L) and the cortex (M–P), stained with anti-GFAP and Alexa 555 secondary antibodies (in red). In untreated animals numerous GFAP positive cells are found in the corpus callosum (I) in contrast to only few GFAP positive cells in the cortex (M). Upon cuprizone treatment reactive astroglia appear in the cortex and in the corpus callosum. At week 4.5 hypertrophic astrocytes are abundantly detected in both areas (J, N). At weeks 5 and 6 astroglia are still presented in large numbers in the corpus callosum and the cortex, however, the shape of astrocytes alters and their processes become thinner (K, L, O, P). For nucleus staining, slides were counterstained by DAPI. (TIF) [file pone.0022623.s002.tif]
