# Supplementary material for: Spatial and Temporal Profiles of Growth Factor Expression during CNS Demyelination Reveal the Dynamics of Repair Priming
Source: PLoS One. 2011 Jul 27;6(7):e22623. doi: 10.1371/journal.pone.0022623 (PMC3144923; doi:10.1371/journal.pone.0022623)
Supplement: Table S1 — TaqMan® Gene Expression Assays (Applied Biosystems, USA) were used to investigate mRNA expression of different growth factors. (DOC) [file pone.0022623.s003.doc]

| Growth Faktor | Assay number |
| --- | --- |
| NGF | Mm00443039_m1 |
| BDNF | Mm01334042_m1 |
| NT-3 | Mm01182924_m1 |
| GDNF | Mm00599849_m1 |
| NRG 1 | Mm00626552_m1 |
| CNTF | Mm00446373_m1 |
| IGF-1 | Mm00439560_m1 |
| TGF-ß1 | Mm00441724_m1 |
| FGF-2 | Mm00433287_m1 |
| HGF | Mm01135184_m1 |
| LIF | Mm00434761_m1 |
| PDGF-A | Mm01205760_m1 |
| EGF | Mm01316968_m1 |
| HPRT | Mm00446968_m1 |

**Table 1**
